# Clinical characteristics, outcomes, and risk factors for mortality in hospitalized patients with COVID-19 and cancer history: a propensity score-matched study

**DOI:** 10.1186/s13027-020-00339-y

**Published:** 2020-12-17

**Authors:** Majid Sorouri, Amir Kasaeian, Helia Mojtabavi, Amir Reza Radmard, Shadi Kolahdoozan, Amir Anushiravani, Bardia Khosravi, Seyed Mohammad Pourabbas, Masoud Eslahi, Azin Sirusbakht, Marjan Khodabakhshi, Fatemeh Motamedi, Fatemeh Azizi, Reza Ghanbari, Zeynab Rajabi, Ali Reza Sima, Soroush Rad, Mohammad Abdollahi

**Affiliations:** 1 Internal Medicine, Digestive Diseases Research Center, Digestive Diseases Research Institute, Shariati Hospital, Tehran University of Medical Sciences, Kargar Shomali Avenue, Tehran, Iran; 2 Hematology, Oncology, and Stem Cell Transplantation Research Center, Shariati Hospital, Tehran University of Medical Sciences, Tehran, Iran; 3 Department of Internal medicine, Shariati hospital, Tehran University of Medical Sciences, Tehran, Iran; 4 Department of Radiology, Shariati Hospital, Tehran University of Medical Sciences, Tehran, Iran; 5 Digestive Oncology Research Center, Digestive Diseases Research Institute, Tehran University of Medical Sciences, Tehran, Iran

**Keywords:** Cancer, COVID-19, Case-control study, Mortality, Severe clinical events, Propensity score matching, Logistic regression, Poisson regression

## Abstract

**Background:**

COVID-19 has caused great concern for patients with underlying medical conditions. We aimed to determine the prognosis of patients with current or previous cancer with either a PCR-confirmed COVID-19 infection or a probable diagnosis according to chest CT scan.

**Methods:**

We conducted a case control study in a referral hospital on confirmed COVID-19 adult patients with and without a history of cancer from February^25th^ to April^21st^, 2020. Patients were matched according to age, gender, and underlying diseases including ischemic heart disease (IHD), diabetes mellitus (DM), and hypertension (HTN). Demographic features, clinical data, comorbidities, symptoms, vital signs, laboratory findings, and chest computed tomography (CT) images have been extracted from patients’ medical records. Multivariable logistic regression was used to estimate odd ratios and 95% confidence intervals of each factor of interest with outcomes.

**Results:**

Fifty-three confirmed COVID-19 patients with history of cancer were recruited and compared with 106 non-cancerous COVID-19 patients as controls. Male to female ratio was 1.33 and 45% were older than 65. Dyspnea and fever were the most common presenting symptoms in our population with 57.86 and 52.83% respectively. Moreover, dyspnea was significantly associated with an increased rate of mortality in the cancer subgroup (*p* = 0.013). Twenty-six patients (49%) survived among the cancer group while 89 patients (84%) survived in control (*p* = 0.000). in cancer group, patients with hematologic cancer had 63% mortality while patients with solid tumors had 37%. multivariate analysis model for survival prediction showed that history of cancer, impaired consciousness level, tachypnea, tachycardia, leukocytosis and thrombocytopenia were associated with an increased risk of death.

**Conclusion:**

In our study, cancer increased the mortality rate and hospital stay of COVID-19 patients and this effect remains significant after adjustment of confounders. Compared to solid tumors, hematologic malignancies have been associated with worse consequences and higher mortality rate. Clinical and para-clinical indicators were not appropriate to predict death in these patients.

## Introduction

The coronavirus disease 2019 (COVID-19) pandemic outbreak, caused by severe acute respiratory syndrome coronavirus 2 (SARS-CoV-2), continues to high levels of mortality and morbidity world-wide [1], especially in elderly patients, patients with underlying chronic illnesses and compromised immune systems and patients with all kinds of cancers [2]. Cancer-specific factors can accentuate the systemic immunosuppressive state in patients, such as antineoplastic therapy, supportive medications like steroids, and the immunosuppressive properties of cancer itself [3–6]. Around 60% of deaths are infection related to cancer patients, especially with underlying hematological malignancies [7].

Recent nationwide cohort and retrospective investigations in China [2, 8] on patients who had been previously diagnosed with cancer (regardless of type or duration of this condition), revealed that the COVID-19 patients with cancer are associated with increased risk for complications as well as high mortality rate (28.6%) [2], compared with non-cancerous COVID-19 patients [9]. However, the result cannot be applied to other countries with different cancer epidemiology and practice [10].

Given the worldwide prevalence of cancer [11] and the high incidence of this novel coronavirus, determining whether COVID-19 patients with a current or past history of hematological malignancy or invasive solid tumor have a poorer prognosis and higher mortality rate could be very important. For this purpose, we conducted a case-control study and analyzed their medical records in Shariati hospital, Tehran, Iran.

## Material and methods

### Study design and participants

We conducted a case-control study on 53 COVID-19 patients with history of cancer and 106 matched non-cancerous COVID-19 patients as controls in a center served as a government designated hospital (Shariati hospital, Tehran, Iran) for adult COVID-19 patients, from February^25th^, 2020, to April^21st^, 2020. The control group were matched in terms of age, gender, underlying disease including ischemic heart disease (IHD), diabetes mellitus (DM), and hypertension (HTN) and hospitalization time. Transplant recipients (kidney, heart, and bone marrow) were excluded from the study due to control this plausible confounder and high rate of mortality in this subgroup of patients.

All demographic features, clinical data, comorbidities, symptoms, vital signs, laboratory findings, and chest computed tomography (CT) images have been extracted from the baseline patients’ medical records. Primary cancer characteristics and detailed treatment information were also obtained by review of the patients’ past medical records.

The study was approved by the ethics committee of Tehran University of Medical Sciences. The ethics committee waived the requirement for informed patient consent for this retrospective study subject to the anonymity of patients.

### Study definitions

COVID-19 was diagnosed according to the World Health Organization (WHO) guideline [12] and confirmed by SARS-CoV-2 RNA using the real-time reverse transcription-polymerase chain reaction (RT-PCR) assay of nasal and/or pharyngeal specimens alongside of chest CT scans. Two attending radiologists reviewed all the chest images, independently.

### Outcome measures

Variables extracted from patients’ medical records appear in Tables 1, 2, 3 and 4.

**Table 1:** Demographics, baseline characteristics and outcome of 159 patients categorized in cancer, and without cancer subgroups and 53 cancerous patients categorized into alive and dead

Variables	W/O cancer *N* = 106 (%)^a^	Cancer *N* = 53 (%)	*p* value	Cancer		*p* value
				Alive *N* = 26 (%)	Dead *N* = 27 (%)	
Age						
< 50	24 (22.6)	8 (15.1)		3 (11.5)	5 (18.5)	
50–65	34 (32.1)	21 (39.6)	0.453	13 (50)	8 (29.6)	0.374
> 65	48 (45.3)	24 (45.3)		10 (38.5)	14 (51.9)	
Sex						
Female	46 (43.4)	22 (41.5)	0.821	14 (53.85)	8 (29.6)	0.098
Male	60 (56.6)	31 (58.5)		12 (46.15)	19 (70.4)	
BMI^b^	25.7 (23.1–29.3)	24.8 (22.4–30.4)	0.528	23.4 (21.9–29.1)	26.6 (23–31.1)	0.180
Past medical history						
DM	16 (15.1)	9 (17)	0.758	4 (15.4)	5 (18.5)	0.990
HTN	30 (28.3)	15 (28.3)	0.990	10 (38.5)	5 (18.5)	0.135
IHD	18 (17)	8 (15.1)	0.762	4 (15.4)	4 (14.8)	0.990
Symptoms:						
Fever	54 (50.9)	30 (56.6)	0.500	13 (50)	17 (63)	0.412
Cough	56 (52.8)	190 (35.9)	0.043	11 (42.3)	8 (29.6)	0.398
Dyspnea	64 (60.4)	28 (52.8)	0.364	9 (34.6)	19 (70.4)	0.013
Chills	4 (3.8)	3 (5.7)	0.585	2 (7.7)	1 (3.7)	0.610
Myalgia	16 (15.1)	8 (15.1)	0.990	4 (15.4)	4 (14.8)	0.990
Nausea	8 (7.6)	15 (28.3)	0.000	10 (38.5)	5 (18.5)	0.135
Diarrhoea	3 (2.8)	1 (1.9)	0.720	1 (3.9)	0	0.491
Sore throat	2 (1.9)	2 (3.8)	0.474	1 (3.9)	1 (3.7)	0.990
Fatigue	35 (33)	26 (49.1)	0.050	12 (46.2)	14 (51.9)	0.786
Anorexia	10 (9.4)	9 (17)	0.167	7 (26.9)	2 (7.4)	0.076
Chest pain	10 (9.4)	1 (1.9)	0.077	1 (3.9)	0	0.304
Signs:						
Low consciousness	7 (6.6)	7 (13.2)	0.166	1 (3.9)	6 (22.2)	0.100
O_2_Saturation						
> 96%	39 (38.6)	18 (34.6)		5 (20)	13 (48.2)	
90–96%	47 (46.5)	22 (42.3)	0.449	12 (48)	10 (37)	0.083
< 90%	15 (14.9)	12 (23.1)		8 (32)	4 (14.8)	
Systolic blood pressure (mmHg)						
< 100	4 (3.9)	9 (17)		4 (15.4)	5 (18.5)	
100–140	75 (73.5)	35 (66)	0.025	17 (65.4)	18 (66.7)	0.990
> 140	23 (22.6)	9 (17)		5 (19.2)	4 (14.8)	
Respiratory rate (/min)						
< 25	87 (86.1)	43 (81.1)	0.416	24 (92.3)	19 (70.4)	0.076
> 25	14 (13.9)	10 (18.9)		2 (7.7)	8 (29.6)	
Pulse Rate (/min)						
< 100	65 (64.4)	29 (55.8)	0.301	19 (73.1)	10 (38.5)	0.025
> 100	36 (35.6)	23 (44.2)		7 (26.9)	16 (61.5)	
Temperature (^o^c)						
< 37.8	63 (61.8)	37 (69.8)		20 (76.9)	17 (63)	
37.8–39	28 (27.4)	12 (22.6)	0.594	5 (19.2)	7 (25.9)	0.493
> 39	11 (10.8)	4 (7.6)		1 (3.9)	3 (11.1)	
qSOFA						
0	68 (64.2)	25 (47.2)		14 (53.9)	11 (40.7)	
1	35 (33)	22 (41.5)		11 (42.3)	11 (40.7)	
2	3 (2.8)	3 (5.7)	0.020	0	3 (11.1)	0.417
3	0	3 (5.7)		1 (3.8)	2 (7.5)	

*BMI* Body mass index, *DM* diabetes mellitus, *HTN* hypertension, *IHD* ischemic heart disease, *qSOFA* quick sequential organ failure assessment

^a^ Percentages were calculated by total, survived, and non-survived patients number as denominator for each column respectively

^b^ median (IQR)

**Table 2:** Laboratory findings and imaging results in 159 patients categorized in cancer, and without cancer subgroups and 53 cancerous patients categorized into alive and dead

	W/O cancer N (%)^a^	Cancer N (%)	*p* value	Cancer		*p* value
				Alive; *N* = 26 (%)	Dead; *N* = 27 (%)	
Covid RT-PCR						
Positive	44 (41.5)	20 (37.7)	0.887	8 (30.8)	12 (44.4)	0.137
Hemoglobin (g/dl)						
< 10	10 (9.4)	24 (45.3)	0.000	10 (38.5)	14 (51.9)	0.404
> 10	86 (81.1)	28 (52.8)		16 (61.5)	12 (44.4)	
Missing	10 (9.4)	1 (1.9)		–	1 (3.7)	
Platelet (/microliter)						
< 150,000	24 (22.6)	29 (54.7)	0.000	10 (38.5)	19 (70.4)	0.025
> 150,000	71 (67)	23 (43.4)		16 (61.5)	7 (25.9)	
Missing	10 (9.4)	1 (1.9)		–	1 (3.7)	
WBC (/microliter)						
< 4000	11 (10.4)	15 (28.3)	0.000	8 (30.8)	7 (25.9)	0.779
4000–12,000	70 (66)	18 (34)		10 (38.4)	8 (29.6)	
> 12,000	15 (14.2)	19 (35.8)		8 (30.8)	11 (40.7)	
Missing	10 (9.4)	1 (1.9)		–	1 (3.7)	
Lymphocyte (/microliter)						
< 1000	34 (32.1)	15 (28.3)	0.082	12 (46.2)	5 (18.5)	0.028
1000–4000	35 (33)	17 (32.1)		10 (38.4)	5 (18.5)	
> 4000	1 (0.9)	4 (7.5)		0	4 (14.8)	
Missing	36 (34)	17 (32.1)		4 (15.4)	13 (48.2)	
Neutrophil (/microliter)						
< 1500	1 (0.9)	7 (13.2)	0.000	5 (19.2)	2 (7.4)	0.908
1500–7700	52 (49.1)	15 (28.3)		9 (34.6)	6 (22.2)	
> 7700	17 (16)	14 (26.4)		8 (30.8)	6 (22.2)	
Missing	36 (34)	17 (32.1)		4 (15.4)	13 (48.2)	
pH						
< 7.25	4 (3.8)	5 (9.4)		1 (3.8)	4 (14.8)	
7.25–7.35	6 (5.7)	6 (11.3)		3 (11.5)	3 (11.1)	
7.35–7.45	65 (61.2)	35 (66.1)	0.372	20 (76.9)	15 (55.6)	0.462
> 7.45	18 (17)	6 (11.3)		2 (7.7)	4 (14.8)	
Missing	13 (12.3)	1 (1.9)		–	1 (3.7)	
PCO_2_ (mmHg)						
< 35	26 (24.5)	16 (30.2)	0.769	5 (19.2)	11 (40.7)	0.008
35–45	41 (38.7)	19 (35.9)		15 (57.7)	4 (14.8)	
> 45	24 (22.6)	14 (26.4)		5 (19.2)	9 (33.3)	
Missing	15 (14.2)	4 (7.5)		1 (3.8)	3 (11.1)	
HCO_3_ (mEq/L)						
< 22	23 (21.7)	21 (39.6)	0.100	6 (23.1)	15 (55.6)	0.018
22–26	30 (28.3)	13 (24.6)		10 (38.4)	3 (11.1)	
> 26	38 (35.8)	15 (28.3)		9 (34.6)	6 (22.2)	
Missing	15 (14.2)	4 (7.5)		1 (3.8)	3 (11.1)	
Na (mEq/L)						
< 135	16 (15.1)	9 (17)	0.739	5 (19.2)	4 (14.8)	0.790
135–145	76 (71.7)	40 (75.5)		19 (73.1)	21 (77.8)	
> 145	3 (2.8)	3 (5.6)		2 (7.7)	1 (3.7)	
Missing	11 (10.4)	1 (1.9)		–	1 (3.7)	
K (mEq/L)						
< 3.5	5 (4.7)	3 (5.6)	0.985	1 (3.8)	2 (7.4)	0.501
3.5–5	76 (71.7)	40 (75.5)		22 (84.6)	18 (66.7)	
> 5	15 (14.2)	8 (15.1)		3 (11.5)	5 (18.5)	
Missing	10 (9.4)	2 (3.8)		–	2 (7.4)	
AST (IU/L)						
< 40	38 (35.8)	14 (26.4)		8 (30.8)	6 (22.2)	
> 40	34 (32.1)	31 (58.5)	0.022	16 (61.5)	15 (55.6)	0.759
Missing	34 (32.1)	8 (15.1)		2 (7.7)	6 (22.2)	
ALT (IU/L)						
< 40	53 (50)	28 (52.8)	0.181	15 (57.7)	13 (48.2)	0.990
> 40	20 (18.9)	18 (34)		9 (34.6)	9 (33.3)	
Missing	33 (31.1)	7 (13.2)		2 (7.7)	5 (18.5)	
ALK-p (IU/L)						
< 200	46 (43.4)	14 (26.4)		7 (26.9)	7 (25.9)	
> 200	24 (22.6)	31 (58.5)	0.000	16 (61.5)	15 (55.6)	0.990
Missing	36 (34)	8 (15.1)		3 (11.5)	5 (18.5)	
PTT (seconds)						
< 45	70 (66)	37 (69.8)		19 (73.1)	18 (66.7)	
> 45	2 (1.9)	3 (5.6)	0.246	3 (11.5)	0	0.238
Missing	34 (32.1)	13 (24.6)		4 (15.4)	9 (33.3)	
INR						
< 1.5	60 (56.6)	26 (49.1)		13 (50)	13 (48.2)	
> 1.5	14 (13.2)	19 (35.8)	0.006	10 (38.4)	9 (33.3)	0.990
Missing	32 (30.2)	8 (15.1)		3 (11.5)	5 (18.5)	
Total Bilirubin (mg/dl)						
< 1.5	47 (44.3)	20 (37.7)		12 (46.1)	8 (29.6)	
> 1.5	7 (6.6)	16 (30.2)	0.001	6 (23.1)	10 (37)	0.315
Missing	52 (49.1)	17 (32.1)		8 (30.8)	9 (33.3)	
Creatinine (mg/dl)						
< 1.2	69 (65.1)	35 (66)		19 (73.1)	16 (59.3)	
> 1.2	28 (26.4)	17 (32.1)	0.628	7 (26.9)	10 (37)	0.375
Missing	9 (8.5)	1 (1.9)		–	1 (3.7)	
CRP^¥^ (mg/L)	68.7 (27; 82)	79 (43; 98)	0.033	70 (19.5;101.5)	79 (76.5;95)	0.282

*WBC* White blood cell, *AST* aspartate aminotransferase, *ALT* alanine aminotransferase, *ALK-P* Alkaline Phosphatase, *PTT* activated **partial thromboplastin** time, *INR* international normalized ratio, *CRP* C-reactive protein, *GGO* ground glass opacity

^a^ Percentages were calculated by the number of total, alive, and dead patients as denominator for each column respectively

**Table 3:** Imaging results in 159 patients categorized in cancer, and without cancer subgroups and 53 cancerous patients categorized into alive and dead

CT findings						
	W/O cancer N (%)	Cancer N (%)	*p* value	Cancer		*p* value
				Alive; *N* = 26 (%)	Dead; *N* = 27 (%)	
Consolidation	45 (42.5)	21 (39.6)	0.368	9 (34.6)	12 (44.4)	0.990
GGO	51 (48.1)	26 (49.1)	0.60	12 (46.2)	14 (51.9)	0.204
Bilateral involvement	51 (48.1)	25 (47.2)	0.334	10 (38.4)	15 (55.6)	0.378
Total CT involvement score^a^	10 (6,12)	7 (4,11.5)	0.005	5.5 (2,9)	6 (2,15)	0.009

^a^Median (IQR) € CT scan was reported in 53 non-cancerous patients, 28 cancer patients (12 alive and 16 dead)

**Table 4:** Outcomes in 53 cancer and 106 control patients. Univariate and multivariate analysis were shown for cancer variable. In multivariate analysis, results were adjusted for o2sat, temperature, respiratory rate, pulse rate, systolic blood pressure, level of consciousness, hemoglobin, Wight blood cells counts, platelet counts and creatinine

	W/O cancer n (%)	Cancer n (%)	*p* value	Univariate analysis		Multivariate analysis	
				Odds ratio (CI95%)	*P* value	Odds ratio (CI95%)	*P* value
Death	17 (16)	27 (50.9)	0.000	5.4 (2.57–11.48)	0.000	3.57 (1.01–12.61)	0.048
ICU admission	28 (26.4)	29 (54.7)	0.000	3.37 (1.68–6.72)	0.001	1.52 (0.56–4.12)	0.406
Intubation	25 (23.6)	27 (50.9)	0.001	3.36 (1.67–6.78)	0.001	1.44 (0.51–4.08)	0.493
Hospital stay; days	10 (6,12)^a^	6 (2,11)^a^	0.005	1.69 (1.49–1.92)^b^	0.000	1.44 (1.24–1.67)^b^	0.000

^a^ Median (IQR)

^b^ *IRR* incidence-rate ratio

To calculate “chest CT-scan total score,” each lung was divided into three zones, (a) upper zone (above the level of carina), (b) middle zone (between the carina and inferior pulmonary vein) and (c) lower zone (below the level of the inferior pulmonary vein). Each zone was evaluated for the percentage of involvement by either of the mentioned parenchymal abnormalities and was assigned a score from 0 to 4, 0: no involvement, 1: 1–25% involved, 2: 26–50% involved, 3: 51–75% involved, 4: 76–100% involved. “Chest CT-scan total score” was calculated by summing the scores from all six zones yielding a score between 0 and 24.

The *qSOFA* score (also known as quickSOFA): using three criteria, assigning one point for low blood pressure (SBP ≤ 100 mmHg), high respiratory rate (≥22 breaths per minute), or altered mentation (Glasgow coma scale< 15).

All of hospitalized adult patients with COVID-19 were treated by lopinavir/ritonavir (kaletra regimen). Antibiotics were appropriately prescribed to treat bacterial infections in eligible participants.

An end-point outcome (that was monitored up to April^21st^, 2020, the final date of follow-up): severe illness requiring admission to an intensive care unit (ICU), the use of invasive mechanical ventilation, days of hospital stay, or in-hospital death.

### Statistical analysis

For descriptive analysis to show the baseline demographic information of the participants included in our analyses, categorical variables were described in frequencies (%). Continuous variables were presented as the mean (standard deviation (SD)) or as the median with interquartile range (IQR), as appropriate. The Shapiro-Wilk test was used to test the normality of data distribution. Parametric and nonparametric tests, including t-test and Mann-Whitney test, were used for comparing quantitative variables and Chi-squared test was applied for comparing categorical variables.

We performed propensity score matching (PSM) using a 1:2 ratio to remove the effect of sex, age, diabetes, hypertension, and ischemic heart disease differences between patients with cancer and patients without cancer. We did this to remove the effects of confounders as much and possible and also needing to check less variables in the multivariable models due to low sample size.

We assessed the effects of several variables on the outcomes of death, ICU admission, and intubation using separate multivariable logistic regression models and a multivariable Poisson regression model (for hospital stay) to estimates the relevant adjusted odds ratios and rate ratio. The variables were chosen based on their clinical importance and their obtainability including level of consciousness, o2sat, systolic blood pressure, temperature, respiratory rate, pulse rate, hemoglobin, white blood cells counts, platelet counts and creatinine. Then, every variable with a *p*-values less than 0.2 in a univariable model entered the multivariable models.

Statistical analyses were performed using Stata (Corp. 2009. Stata Statistical Software: Release 11. College Station, TX: StataCorp LP.) and the R package MatchIt. Statistical significance was considered as a two-side *P*-value less than 0.05.

## Result

Fifty-three patients with confirmed COVID-19 and history of cancer were recruited and compared with 106 non-cancerous confirmed COVID-19 patients. Demographics, baseline characteristics, presenting symptoms, past medical history, drug history and ultimate outcome of the total cancerous and non-cancerous patients are summarized in Table 1. Male to female ratio of our study patients was 1.33 and around 45% of all patients were older than 65. Dyspnea and fever were among the most common presenting symptoms in our population with 57.86 and 52.83% respectively. Moreover, dyspnea was significantly associated with an increased rate of mortality in the cancer group (*p* = 0.013). The most prevalent comorbidity in COVID-19 patients with cancer was HTN (28.30%).

Patients with cancer had a statistically significant higher qSOFA score. Although SBP was lower in the cancer group significantly (*p* = 0.025), but mortality did not increase.

Table 2 shows laboratory findings in cancer and non-cancer patients with COVID-19. Pancytopenia was observed significantly more often in the cancer population (*p* = 0.000), however, only thrombocytopenia increased the rate of death among this group. The cancer group has a higher CRP titer, which did not lead to an increase in mortality. Regarding liver function, total bilirubin and INR showed higher levels in cancer patients, however mortality was not affected. Most common CT findings were ground glass opacities and bilateral involvement.

Death rates, ICU admission, intubation and length of hospital stay were significantly higher among COVID-19 patients with cancer, which is illustrated with detail in Table 4. The average length of hospital stay was 6 days in cancerous patients, and 10 days in the non-cancer population (*p* = 0.005). Twenty-six patients (49%) survived among the cancer group while 89 patients (84%) survived in the control (*p* = 0.000). ICU admission rates among the cancer group was 54.71%, while in the non-cancer group it was 26.41%. Tracheal intubation was done for 50.94% versus 23.58% in cancer and non-cancer patients, respectively. Both ICU admission and intubation rates were statistically significant in our cancer population (*p* = 0.000 and 0.001 respectively).

Table 5 focuses on major outcomes in specific cancer types, including solid tumors and hematologic cancers. Hematologic cancers seem to have far worse consequences than solid tumors. Finally, multivariate analysis model for survival prediction showed that history of cancer, impaired consciousness level, tachypnea, tachycardia, leukocytosis and thrombocytopenia were associated with an increased risk of death which is presented in Appendix.

**Table 5:** Main outcomes in different types of cancer

Cancer types	Total *N* = 53 (%)^b^	Dead *N* = 27 (%)^c^	ICU Admission *N* = 29(%)^d^	Intubation *N* = 27 (%)^e^	Hospital stay; days Median (IQR)
Solid tumors	29 (54.7)	10 (37)	11 (37.9)	10 (37)	6 (2;10)
Gastrointestinal	14 (26.4)	5 (18.5)	6 (20.7)	5 (18.5)	5.5 (2;11)
Breast	4 (7.6)	2 (7.4)	2 (6.9)	2 (7.4)	5 (2.5;7.5)
Lung	5 (9.4)	0	0	0	7 (6;13)
Genitourinary	5 (9.4)	2 (7.4)	2 (6.9)	2 (7.4)	8 (2;17)
Laryngeal	1 (1.9)	1 (3.7)	1 (3.5)	1 (3.7)	1
Hematologic cancers	24 (45.28)	17 (63) ^a^	18 (62.1)	17 (63)	6.5 (2;14)
ALL	3 (5.66)	2 (7.4)	2 (6.9)	2 (7.4)	7 (1;21)
AML	9 (16.98)	5 (18.5)	5 (17.2)	5 (18.5)	6 (2;13)
CLL	4 (7.55)	4 (14.8)	4 (13.8)	4 (14.8)	4 (1.5;15.5)
CML	1 (1.89)	1 (3.7)	1 (3.5)	1 (3.7)	10
Lymphoma	5 (9.43)	4 (14.8)	4 (13.8)	4 (14.8)	9 (8;15)
MM	2 (3.77)	1 (3.7)	2 (6.9)	1 (3.7)	5 (1;9)

*ALL* Acute lymphoblastic leukemia, *AML* Acute myeloid leukemia, *CLL* Chronic lymphocytic leukemia, *CML* Chronic myelogenous leukemia, *MM* Multiple myeloma

^a^ Mortality was significantly higher in hematologic cancer patients than patients with solid tumor (*P* value = 0.013)

^b^Percentage in 53 cancer patients

^c^Percentage in 27 non-survived cancer patients

^d^Percentage in 29 ICU admitted cancer patients

^e^Percentage in 27 intubated cancer patients

## Discussion

In the present study we compared 53 COVID-19 patients with cancer and 106 matched non-cancerous COVID-19 patients. The Propensity Score model was used to determine the distribution of age and sex, hypertension, diabetes and ischemic heart disease, among cancer and non-cancer patients, which were evenly distributed. In addition, in cancer patients these variables were not associated with increased rate of death. Our findings was similar to Menge et al. study, in which 109 cancer and 327 non-cancerous patients have been compared based on age, sex, history of diabetes, hypertension, and ischemic heart disease; no difference observed between alive and dead subgroup [13]. In another report of 28 patients with solid tumors, age and sex had no effect on the outcomes [7]. In several related previous studies, older age and being male were reported as risk factor for mortality in cancerous patients [14–17], while some other studied revealed that hypertension or ischemic heart disease increased the risk of death [16, 17]. These different results may be due to differences in the gender distribution of cancers such as breast or prostate, or in leukemia which are more common at certain ages.

In our study, fever, fatigue, and nausea were more common in cancer patients, but cough was more common in those without cancer. Most studies reporting symptoms of COVID-19 in patients with a history of cancer have described fever, cough, and fatigue as the most common symptoms, although most did not have a control group for comparison [7, 14, 16, 17]. In the present study, dyspnea was the only symptom that was significantly more common in the dead subgroup of cancer patients. This finding was consistent with the reports of Yang et al. [14] and Lee et al. [17]. Although in our study, low blood pressure and high qSOFA scores were more common in cancer patients, alive and dead subgroups had the same scores in this regards. Indicators such as on admission qSOFA did not appear to predict death within cancer patients. The only vital sign significantly higher in the dead subgroup of cancer patients was pulse rate, while its reason is not clear due to a large amount of missing data on concomitant carditis in our patients. However, Yang et al. [14] reported a greater severity of COVID-19 and higher respiratory rate and lower oxygen saturation in the dead subgroup of 205 cancer patients. Lee et al. [17] also reported a higher severity of COVID-19 in the dead subgroup of 800 patients with active cancer.

We observed abnormal CBC, increased liver enzymes, and high CRP occur more commonly in cancer patients than in the control group. In addition, thrombocytopenia, abnormal lymphocyte counts, and abnormal blood gas level were the most common findings in the dead subgroup of cancer patients. Based on our observation CBC has higher predictive value in COVID-19 patients’ outcome (Table 2 and Appendix). This finding is consistent with other studies [13, 14].

In terms of imaging findings, we found the ground-glass opacity and bilateral infiltration to be the most common CT findings in cancer patients, same as L Zhang et al. [7] and Kunyu Yang et al. [14]. In our study, the extent of lung involvement measured with “Total CT involvement score” was the only imaging variable that differed significantly between cancer and noncancerous groups, and also between the dead and alive subgroups of cancerous patients. The extent of involvement in the dead cancer subgroup was higher than the alive subgroup, but it was interesting to note that the extent of lung involvement in cancer patients was lower than the control group. We found few studies that reported the extent of lung involvement in cancer patients. Liang et al. [8] reported the severity of lung involvement in a cohort of 1590 patients, which 18 of whom had cancer, and the severity of CT involvement in cancer patients was more severe than non-cancerous patients. We think it makes sense for the “Total CT involvement score” to be associated with death as an indicator of the severity of COVID-19 disease in groups of cancer patients. However, lower prevalence of cough in cancer patients than in the control group along with the lower CT scores, is hypothesized to be due to weakened immunity which indicates lesser infiltration of the lungs.

The main finding of our study was the increased probability of death in COVID-19 patients with cancer history compared with non-cancerous group, and this effect remains significant after adjustment of confounders. The effect of cancer on ICU admission and intubation were not significant after adjustment of confounders. In a related study performed by Menge et al. [13] 109 cancer patients with COVID-19 was compared with 327 non-cancer patients, and they observed that cancer significantly increased the risk of intubation and death but had no effect on the length of hospital stay. Lower duration of hospitalization in the cancer group is mainly due to the higher incidence of death within this population, mostly within the hematologic cancer subgroup, in comparison to the other cancers subgroups and non-cancerous group.

Our study revealed that COVID-19 patients with hematologic cancers had the highest risk of death, ICU admission and intubation that was consistent with other studies comparing patients with hematologic cancer with solid tumors [13, 14]. The death of patients in the early days of hospitalization may have hidden the effect of cancer on hospital stay in Menge’s study [13] and also the hematologic cancers subgroup. In a recent meta-analysis on the survival of cancer patients affected with COVID-19 death rate in 24 cancer patients was 20% comparing to the 1393 non cancer patients which was 7.4% with odds ratio of 2.25 and *p* = 0.016 [18]. Many factors could attribute to the increased risk of death in the hematologic population. Lower neutrophil counts prone this population to many bacterial, viral, and fungal super-infections which expectantly rises the risk of further complication and mortality. Moreover, thrombocytopenia is associated with ICU admission, mechanical ventilation or death within the first 14 days of admission with an OR of 2.48, in similar COVID-19 reports [19]. Prevalence of thrombocytopenia is higher within the hematologic cancer population on the admission, this could solely result is severe outcomes and death in addition to contraindication for anticoagulation prescription in this population which is considered a potential therapeutic option in severe COVID-19 cases [20].

Lack of analysis and missing data on the spread and staging of cancers and their therapies was the most limitation in our study. In addition, due to the small number of patients in the subgroups of different types of cancer, we were not able to use a statistical model. Matching with a control group and reporting different clinical and paraclinical variables are the strengths of our study.

In summary, we reported clinical and paraclinical variables in cancer and non-cancer patients with COVID-19 and compared them regarding the different patients’ outcome. Despite the fact that cancer patients are worse off on admission, clinical and paraclinical indicators are not appropriate to predict death in these patients. Cancer significantly increases the risk of adverse outcomes, and hematologic cancers have a higher risk compared to solid tumors.

## Appendix

**Table 6:** Univariate and multivariate analysis model for survival prediction

Variables	Univariate analysis		Multivariate analysis	
	Odds ratio (CI95%)	***P*** value	Odds ratio (CI95%)	***P*** value
Cancer	5.4 (2.57–11.48)	0.000	3.57 (1.01–12.61)	0.048
Level of consciousness				
Awareness	Ref.		Ref.	
Non awareness	12.44 (3.28–47.26)	0.000	12.45 (1.82–85)	0.010
O_2_Saturation				
> 95%	Ref.		Ref.	
90–95%	0.81 (0.27–2.42)	0.709	1.49 (0.35–6.40)	0.595
< 90%	2.73 (0.96–7.79)	0.060	2.13 (0.47–9.64)	0.328
Systolic blood pressure (mmHg)				
100–140	Ref.		Ref.	
< 100	2.18 (0.68–7.02)	0.189	0.57 (0.09–3.63)	0.553
> 140	0.71 (0.28–1.82)	0.479	0.80 (0.18–3.57)	0.772
Temperature (^o^c)				
< 37.8	Ref.		Ref.	
37.8–39	0.82 (0.35–1.88)	0.634	–	
> 39	1.22 (0.38–3.89)	0.732	–	
Respiratory rate (/min)				
< 25	Ref.		Ref.	
> 25	7.29 (2.83–18.77)	0.000	8.85 (2.26–34.71)	0.002
Pulse rate (/min)				
< 100	Ref.		Ref.	
> 100	4. 11 (1.96–8.64)	0.000	6.08 (1.98–18.69)	0.002
Hemoglobin (g/dl)				
> 10	Ref.		Ref.	
< 10	4.22 (1.88–9.48)	0.000	1.56 (0.40–5.98)	0.520
WBC (/microliter)				
4000–12,000	Ref.		Ref.	
< 4000	1.86 (0.69–4.98)	0.219	0.53 (0.11–2.55)	0.431
> 12,000	4.18 (1.78–9.83)	0.001	4.19 (1.12–15.76)	0.034
Platelet (/microliter)				
> 150,000	Ref.		Ref.	
< 150,000	3.49 (1.66–7.37)	0.001	7.32 (2.11–25.38)	0.002
Creatinine (mg/dl)				
< 1.2	Ref.		Ref.	
> 1.2	2.22 (1.05–4.71)	0.037	1.44 (0.41–4.93)	0.564
